# Salmonella Serotype Enteritidis Neck Abscess Without Associated Gastrointestinal Illness: A Case Report

**DOI:** 10.7759/cureus.101840

**Published:** 2026-01-19

**Authors:** Quan Lu, Donald Dumford

**Affiliations:** 1 Otolaryngology, Northeast Ohio Medical University, Rootstown, USA; 2 Infectious Disease, Cleveland Clinic Akron General, Akron, USA; 3 Internal Medicine, Northeast Ohio Medical University, Rootstown, USA

**Keywords:** extraintestinal salmonellosis, infection risk factors, infectious disease, neck abscess, salmonella infection

## Abstract

Salmonella infection most often manifests in the gastrointestinal tract, leading to diarrheal illness. Extraintestinal salmonellosis is an uncommon occurrence, especially in head and neck infections and abscesses. We present an unusual case of *Salmonella enterica *serovar*Enteritidis* neck abscess in a 56-year-old female without concurrent gastroenteritis complicated by necrotic eschar development, requiring prolonged antibiotic therapy, corticosteroid taper, and surgical debridement. The report highlights the importance of close follow-up with a low threshold for extension of antibiotic duration, and surgical intervention if no improvement, clinical deterioration, or development of skin complications to promote optimal outcomes in neck abscesses.

## Introduction

*Salmonella* infection presents in one of five clinical forms, including gastroenteritis, bacteremia, focal infection, enteric or typhoid fever, and a chronic carrier state. Infection occurs after ingestion of the organism through contaminated food or water. Extraintestinal salmonellosis, causing focal infection resulting from hematogenous spread outside the gastrointestinal tract, is rare, accounting for approximately 8% of all episodes, and can involve the urinary tract, lungs or pleural space, intra-abdominal sites, the central nervous system, and osteoarticular structures [1]. A neck abscess is an infrequent manifestation of extraintestinal salmonellosis, with only a small number of case reports in the literature. Head and neck infections and abscesses are usually caused by *Staphylococcus, Streptococcus, Haemophilus*, and oral anaerobes [2]. We present a case of a *Salmonella enterica* serotype (serovar) *Enteritidis* neck abscess occurring without concurrent or prior documented gastroenteritis or bacteremia, complicated by necrotic eschar development and requiring prolonged antibiotic therapy, a corticosteroid taper, and surgical debridement. This report emphasizes the importance of considering atypical pathogens in patients with relevant risk factors or in those who do not respond to empiric therapy, employing culture-directed antibiotic therapy, ensuring close follow-up with a low threshold for extending treatment duration, and pursuing surgical intervention in cases of no improvement, clinical deterioration, or development of skin complications to achieve optimal outcomes in neck abscesses.

## Case presentation

In August of 2024, a 56-year-old female with a past medical history significant for uncontrolled type two diabetes mellitus (DM2) with a HbA1c on presentation of 11.3%, hypertension, generalized anxiety disorder, bipolar affective disorder, and chronic obstructive pulmonary disease (COPD), presented with chief complaints of worsening left neck swelling and pain. The neck swelling had begun approximately three weeks before presentation as a nodule without an inciting event, trauma, insect, or animal bite. The nodule had become progressively larger and more painful. The pain had radiated to other areas of her neck and upper back, and begun to drain fluid. Additionally, she had developed subjective fever, chills, and dysphagia.

The patient had initially sought care from her primary care physician three days before admission and had been prescribed a course of oral amoxicillin-clavulanic acid (875/125mg two times per day) and doxycycline (100 mg oral two times per day). There had been no improvement, so she had been advised to go to the emergency department for further immediate imaging. Further review revealed that she had not experienced any recent gastroenteritis symptoms or history of gallbladder condition, had not recently consumed chicken or fish, and had no travel history, environmental, or animal contact. She had a pet dog. She also had not undergone any recent dental procedures, but noted a history of poor oral dentition. Her other surgical history included sigmoidectomy reduction for diverticulitis and appendectomy 11 years ago.

**Figure 1:**
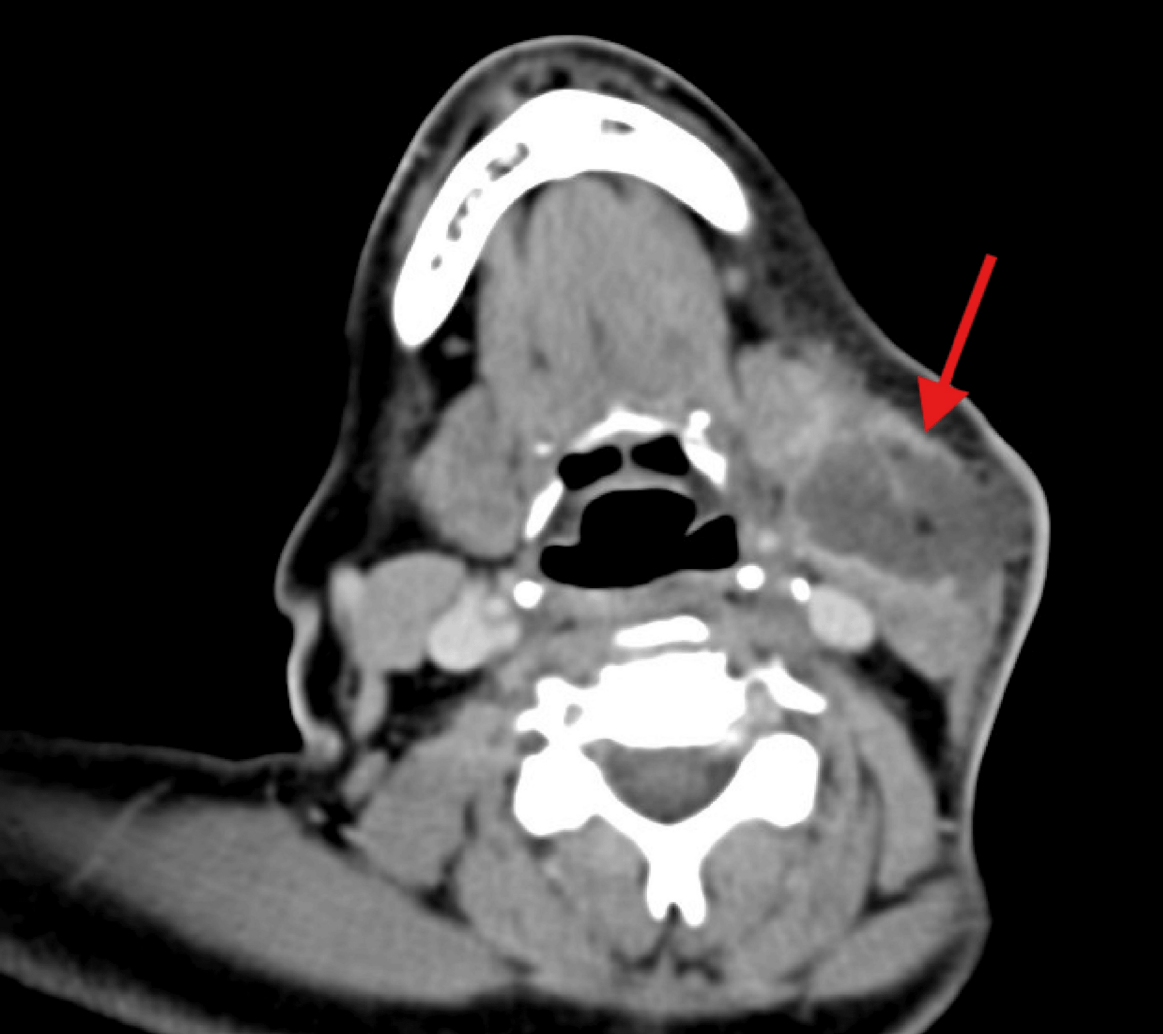
CT image showing Salmonella abscess of the left side of the neck at the suprahyoid and infrahyoid level CT: computed tomography

On examination, a 4.0 x 3.0 cm mass was found in the anterior cervical region of the left neck, with erythematous skin, central early skin necrosis, and tenderness to palpation. Laboratory analysis revealed an elevated leukocyte count of 17,000/uL, with an elevated absolute neutrophil count (ANC) of 13,880/uL, C-reactive protein (CRP) concentration at 23.5 mg/dL, and erythrocyte sedimentation rate (ESR) of 91 mm/hr. HIV screening was negative, initial glucose on basic metabolic panel was 406mg/dL, and HbA1c level was 11.3%. A CT neck soft tissue scan with intravenous (IV) contrast found a 5.0 x 3.2 x 3.5 cm irregular rim-enhancing fluid collection at the suprahyoid and infrahyoid level of the left neck with soft tissue edema and skin thickening. There were also enlarged lymph nodes at level Ib, IIa, and IIb nodal stations on the left side (Figure 1). Blood cultures showed no growth. Table 1 summarizes the pertinent laboratory results at the time of presentation.

**Table 1:** Pertinent laboratory results at time of presentation with Salmonella neck abscess

Test	Result	Reference range
Hemoglobin A1c	11.3%	4.3-5.6%
White blood cell count	17,000 cells/µL	3,700-11,000 cells/µL
Absolute neutrophil count	13,880 cells/uL	1,450-7,500 cells/µL
C-reactive protein	23.5 mg/dL	<0.9 mg/dL
Erythrocyte sedimentation rate	91 mm/hr	0-20 mm/hr
HIV 1/2 combo (Ag/Ab)	Nonreactive	Nonreactive

The patient was initially treated with IV vancomycin (1.25 grams every 12 hours) and ampicillin-sulbactam (3 grams every six hours). Otolaryngology (ENT) initiated IV dexamethasone (4 mg every six hours) and performed a bedside aspiration of the abscess, which produced a small amount of pus that was sent for culture. Oral Maxillofacial Surgery (OMFS) was also consulted, and they determined that the abscess appeared non-odontogenic in origin. The patient’s condition improved with the administration of steroids and antimicrobial therapy. While on steroids as an inpatient, her blood glucose levels fluctuated widely, ranging between 76 and 478 mg/dL, with no appreciable upward trend after dexamethasone was started. Her hyperglycemia was controlled by continuing insulin glargine (initially dosed at 60 units twice daily, then increased to 65 units twice daily) and insulin lispro (10 units with meals plus additional units based on a sliding scale). Although there was concern that corticosteroids could worsen her glucose control, the benefit was felt to outweigh the risk.

A *Salmonella* species was identified from her abscess culture via matrix-assisted laser desorption/ionization time-of-flight mass spectrometry (MALDI-TOF). Serotyping was performed by the state health department, identifying *Salmonella enterica* serotype Enteritidis. Susceptibility testing was performed using the BD Phoenix Automated Microbiology System (BD Diagnostics, Sparks, MD) and reported according to CLSI guidelines for extraintestinal sites. The isolate was found to be susceptible to ampicillin, ceftriaxone, trimethoprim-sulfamethoxazole (TMP/SMX), and ciprofloxacin. Blood cultures demonstrated no growth after five days. Antimicrobial therapy was de-escalated to IV levofloxacin (750 mg daily), and the patient was discharged on oral levofloxacin (750 mg once daily) for 14 days, along with an oral dexamethasone taper (2 mg tablets, two tablets twice daily for three days, one tablet twice daily for two days, and one tablet daily for two days), with outpatient follow-up arranged with ENT and Infectious Disease (ID).

**Figure 2:**
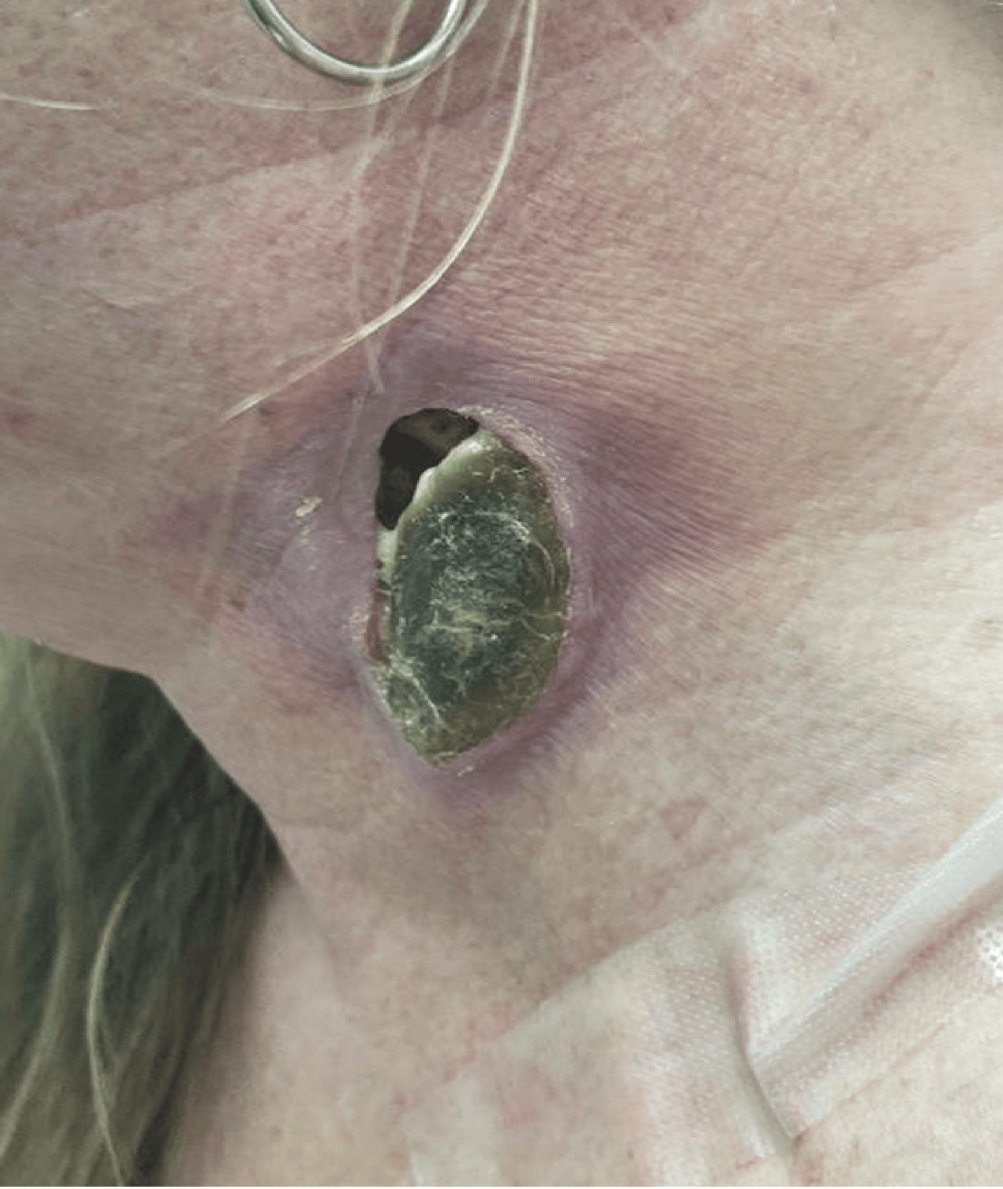
Appearance of Salmonella left neck abscess site at day 21 of antibiotic therapy

At her three-week post-admission follow-up with ID, she continued to experience significant pain in her left neck but denied fever, chills, or night sweats. On examination, the 5.0 x 2.0 cm wound remained open, approximately 90% covered by necrotic eschar, with a small amount of purulent drainage from an open area superiorly and surrounding erythema (Figure 2). A new wound culture was obtained and grew one colony of *Salmonella* species, negative for anaerobes. Her diabetes remained poorly controlled, with a glucose of 269 mg/dL recorded in her comprehensive metabolic panel. She was restarted on oral levofloxacin (750 mg once daily) for an additional 14 days, and ENT proceeded with surgical debridement. At a follow-up visit in November 2024, the patient’s symptoms had completely resolved, and the wound had healed. At subsequent follow-up visits in December 2024 and April 2025, there were no signs or symptoms of recurrence.

## Discussion

There have been only a few reported cases of neck abscesses attributed to *Salmonella enterica* serovar *Enteritidis* in the literature in English. *Salmonella enterica* serovar *Enteritidis* has also been reported to cause parotid and thyroid abscesses [3]. The majority of cases without an underlying carrier state resolved following incision and drainage of the abscess and/or a course of appropriate antibiotics, including fluoroquinolones, cephalexin, cefuroxime, and metronidazole, typically for about 14 days [4-6]. Because this organism is uncommon in this anatomical location, the patient initially received an inappropriate outpatient antibiotic, amoxicillin/clavulanic acid, highlighting the importance of obtaining a culture when possible. A case report of a patient with DM and severe oral disease described treatment with tosufloxacin for four weeks, after the bacteria were no longer isolated following the first two weeks of therapy [7].

Our case is notable in that the patient's wound continued to grow *Salmonella* after two weeks of appropriate antibiotic therapy and required a prolonged four-week course of levofloxacin to completely eradicate the infection. There are two reported cases of necrotizing fasciitis as a complication of *Salmonella enterica* serovar *Enteritidis* neck abscess, both requiring multiple surgical debridements, and one case required flap reconstruction of the defect along with IV ciprofloxacin [8]. The prior literature and this patient's case emphasize the importance of early and adequate source control, which may have contributed to a faster resolution of her infection and a shorter overall treatment duration.

It is unclear what mechanisms led to our patient acquiring and developing a *Salmonella* infection, despite the lack of exposure to known carriers, trauma to the area, or gastrointestinal symptoms. Although we would typically expect the infection to originate in the gastrointestinal tract with subsequent hematogenous dissemination to the site of infection, dissemination beyond the gastrointestinal tract can occur through spread to regional lymphatics followed by dissemination via the thoracic duct [9]. Our patient is similar to several other reported cases, as she had uncontrolled diabetes mellitus with associated immunocompromise, increasing the risk for complicated disease and neck abscess formation [4,5,7,9,10]. A prior case-control study demonstrated an increased risk of *Salmonella* infection, with an odds ratio of 3.1 (95% CI: 1.1-8.6) [11].

Other cases have suggested that additional mechanisms of immune dysregulation, such as chronic liver disease or malignancy, may serve as predisposing factors [4-7,12]. A proposed mechanism for diabetes mellitus (DM) as a risk factor for Salmonella bacteremia includes decreased gastric acidity and prolonged gastrointestinal transit time due to reduced motility from diabetic autonomic neuropathy [11]. Additionally, the effects of uncontrolled diabetes on the immune system further increase the risk of infection. There have been two reported cases in the literature of patients with DM and dental disease presenting with submandibular and lymphatic abscesses caused by *Salmonella enterica* serovar *Enteritidis* [7,10].

## Conclusions

In patients with uncontrolled diabetes or other causes of immune dysregulation who present with neck abscesses unresponsive to standard antibiotics, *Salmonella* species should be considered as a potential causative agent. These patients should receive adequate incision and drainage and empiric antibiotic therapy, with culture of the site to identify the causative organism and guide antibiotic selection. Corticosteroid dosing and tapering may be considered for symptom management. Close follow-up is essential, with a low threshold to extend the duration of antibiotics or pursue surgical intervention if there is no improvement, clinical deterioration, or development of skin complications, in order to achieve the best possible outcome.
